# Supplementary material for: Reduction in SOCE and Associated Aggregation in Platelets from Mice with Platelet-Specific Deletion of Orai1
Source: Cells. 2022 Oct 14;11(20):3225. doi: 10.3390/cells11203225 (PMC9600098; doi:10.3390/cells11203225)
Supplement: Supplementary file 1 [file cells-11-03225-s001.zip › cells-1726371-supplementary.pdf]

SUPPLEMENTARY MATERIAL

Reduction in SOCE and associated aggregation in platelets from mice with platelet-specific deletion of Orai1

Linlin Yang [1,2,§], Roger Ottenheijm [1,2,§], Paul Worley [3], Marc Freichel [1,2,*] and Juan E. Camacho Londoño [1,2,*]

[1] Affiliation 1; Pharmakologisches Institut, Ruprecht-Karls-Universität Heidelberg, INF 366, 69120 Heidelberg, Germany; linlin.yang@pharma.uni-heidelberg.de (L.Y.); roger.ottenheijm@pharma.uni-heidelberg.de (R.O.)

[2] DZHK (German Centre for Cardiovascular Research), partner site Heidelberg/Mannheim, 69120, Germany

[3] The Solomon H. Snyder Department of Neuroscience, Johns Hopkins University, School of Medicine, MD 21205 Baltimore, USA; pworley@jhmi.edu (P.W.)

§ Equally contributed

* Correspondence: juan.londono@pharma.uni-heidelberg.de (J.E.C.L.) and Marc Freichel (marc.freichel@pharma.uni-heidelberg.de); INF-366, D-69120 Heidelberg, Germany, Tel.: +49-6221-54-86861, Fax: +49-6221-54-8644 (J.E.C.L. and M.F.)

Figure S1. CRP-, U46619- or Thrombin-induced platelet aggregation in Orai1 deficient platelets in the presence of ASA and/or Apyrase.

Figure S2. Epinephrine- or arachidonic acid-induced platelet aggregation in Orai1 deficient platelets.

Figure S3. Impaired thapsigargin-induced platelet aggregation in Orai1 deficient platelets.

Supplementary Figures

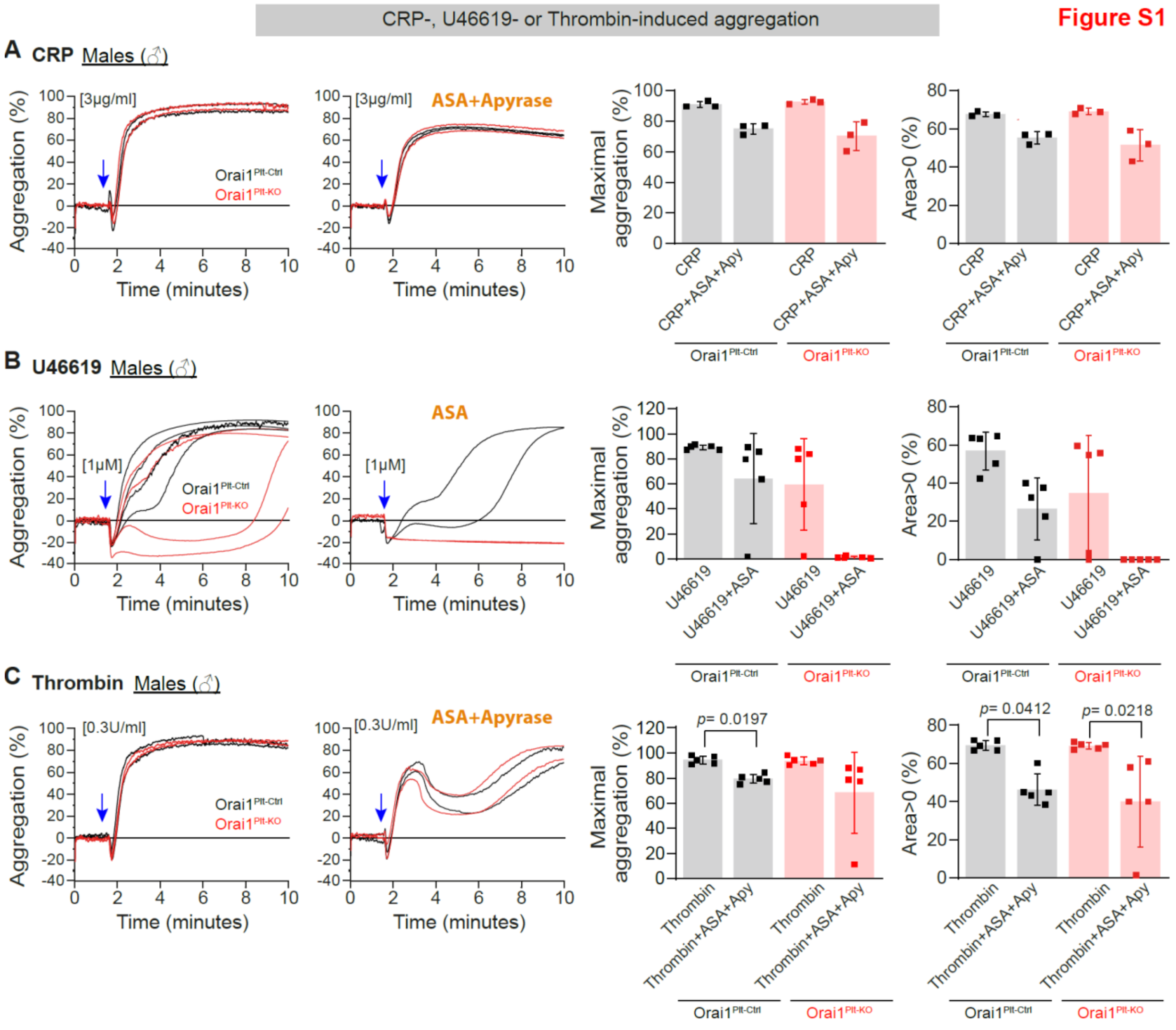

Figure S1. CRP-, U46619- or Thrombin-induced platelet aggregation in Orai1 deficient platelets in the presence of ASA and/or Apyrase. CRP-, U46619- or Thrombin-induced platelet aggregation in the presence of ASA and/or Apyrase analysed in washed platelets from Orai1$^{Plt\text{-}Ctrl}$ (black) and Orai1$^{Plt\text{-}KO}$ (red) male mice is shown (**A-C**). Representative original aggregation traces (left panels), analysis of maximal aggregation (middle right panels) and area under the aggregation curve (right panels) after stimulation with 3µg/ml CRP (**A**), 1µM U46619 (**B**) or 0,3U/ml thrombin (**C**). Further analysis of platelet aggregation in the presence of 1mM acetylsalicylic acid ASA and/or 10U/ml Apyrase, to prevent secondary signalling by TxA2 and secreted ADP, respectively, is also depicted for each agonist. Each dot in (**A, B** and **D**) corresponds to one independent platelet preparation. Blue arrows indicate the agonist stimulation time point. Error bars indicate SD. *p*-values were calculated according to Kruskal-Wallis followed by Dunn's test. CRP: Collagen related peptide; ASA: acetylsalicylic acid; Apy: Apyrase.

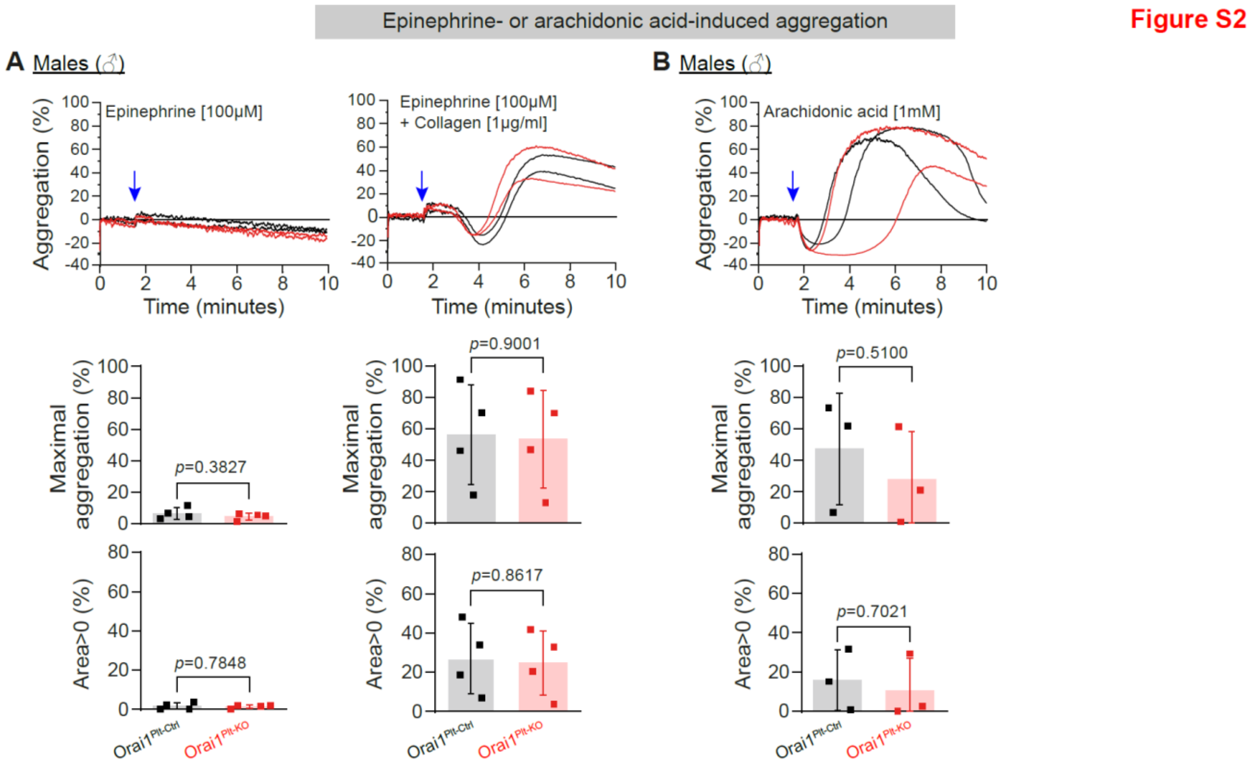

Figure S2. Epinephrine- or arachidonic acid-induced platelet aggregation in Orai1 deficient platelets. Epinephrine- or arachidonic acid-induced platelet aggregation analysed in washed platelets from Orai1$^{Plt\text{-}Ctrl}$ (black) and Orai1$^{Plt\text{-}KO}$ (red) male mice is shown (**A-B**). Representative original aggregation traces (upper panels), analysis of maximal aggregation (middle panels) and area under the aggregation curve (lower panels) after stimulation with 100µM Epinephrine alone or together with 1µg/ml collagen (**A**) or 1mM arachidonic acid (**B**). Each dot in (**A** and **B**) corresponds to one independent platelet preparation. Blue arrows indicate the agonist stimulation time point. Error bars indicate SD. *p*-values were calculated according to unpaired Student's *t*-test.

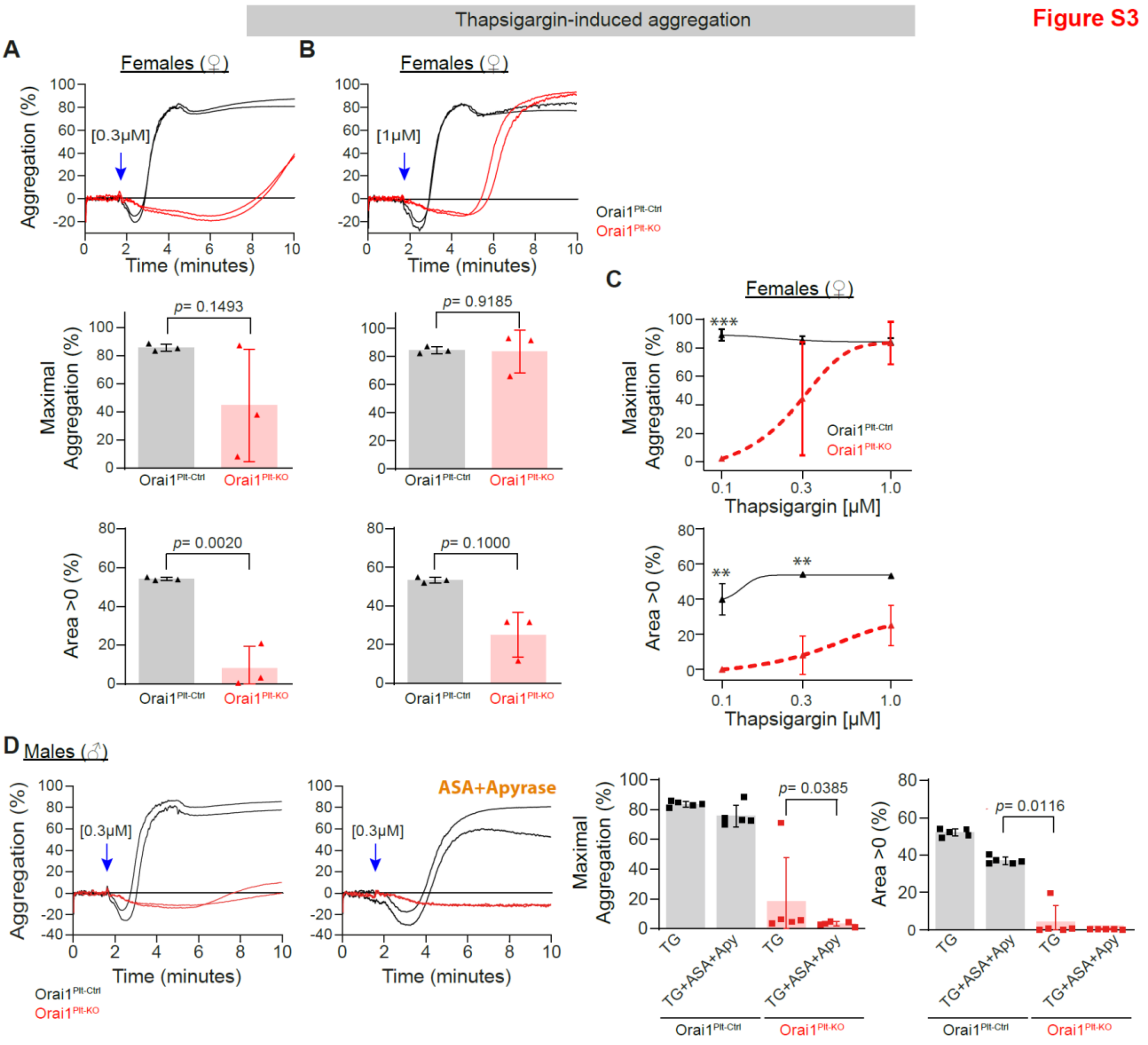

Figure S3. Impaired thapsigargin-induced platelet aggregation in Orai1 deficient platelets. Thapsigargin-induced platelet aggregation in washed platelets from Orai1$^{Plt-Ctrl}$ (black) and Orai1^{Plt-KO} (red) female mice was analysed (**A**-**C**). Representative original aggregation traces (upper panels), analysis of maximal aggregation (middle panels) and area under the aggregation curve (lower panels) after stimulation with 0.3μM (**A**) or 1μM (**B**) thapsigargin. Dose-response curves for the maximal aggregation (upper panel) and the area under the aggregation curve (lower panel) are shown (**C**). Further analysis of Thapsigargin-induced platelet aggregation (male platelets) in the presence of 1mM acetylsalicylic acid ASA and 10U/ml Apyrase, to prevent secondary signalling by TxA2 and secreted ADP, respectively, is shown (n=5) (**D**), Each dot in (**A, B** and **D**) corresponds to one independent platelet preparation. In (C) numbers of independent platelet preparations per thapsigargin concentration were: 0.1μM=3, 0.3μM=3, 1μM=3. Blue arrows indicate the agonist stimulation time point. Error bars indicate SD. *p*-values, **p*<0.05, ***p* <0.01 and ****p* <0.001 were calculated according to the unpaired Student's *t*-test (all panels in A and B except for Area>0 of TG 1μM), Mann-Whitney U test (Area>0 of TG 1μM) or Kruskal-Wallis followed by Dunn's test (Panel D) after Shapiro−Wilk normality test. TG: Thapsigargin. ASA: acetylsalicylic acid; Apy: Apyrase.
